# Functional Roles of Exosomes in Allergic Contact Dermatitis

**DOI:** 10.4014/jmb.2206.06024

**Published:** 2022-09-12

**Authors:** Bocui Song, Qian Chen, Yuqi Li, Shuang Zhan, Rui Zhao, Xue Shen, Min Liu, Chunyu Tong

**Affiliations:** 1 Department of Pharmaceutical Engineering, College of Life Science and Technology, Heilongjiang Bayi Agricultural University, Daqing 163319, Heilongjiang Province, P.R. China; 2 Molecular Mechanism of Disease and Research and Development of Bioactive Substances, College of Life Science and Technology, Heilongjiang Bayi Agricultural University, Daqing 163319, Heilongjiang Province, P.R. China; 3 Animal Husbandry and Veterinary Station of Yongji Economic Development Zone, Jilin 132200, Jilin Province, P.R. China; 4 Department of Biological Science, College of Life Science and Technology, Heilongjiang Bayi Agricultural University, Daqing 163319, Heilongjiang Province, P.R. China

**Keywords:** Allergic contact dermatitis, exosomes, immunoregulation, inflammation

## Abstract

Allergic contact dermatitis (ACD) is an allergen-specific T-cell-mediated inflammatory response, albeit with unclear pathogenesis. Exosomes are nanoscale extracellular vesicles secreted by several cell types and widely distributed in various biological fluids. Exosomes affect the occurrence and development of ACD through immunoregulation among other ways. Nevertheless, the role of exosomes in ACD warrants further clarification. This review examines the progress of research into exosomes and their involvement in the pathogenesis, diagnosis, and treatment of ACD and provides ideas for exploring new diagnostic and treatment methods for this disease.

## Introduction

Exosomes are special extracellular vesicles with a diameter of 40-160 nm, depending on their origin site and the cell lipid bilayer structure. Exosomes from different sources vary in their functions, contents, and sizes [1]. The biophysical properties of exosomes, such as the stiffness and charge, also differ [2]. Studies have found that exosomes of the same cell source have different sizes and morphologies, separating them into nine categories as follows: single vesicle, double vesicle, triple vesicle or more, small double vesicle, oval vesicle, small tubule, large tubule, incomplete vesicle, and pleomorphic vesicle [3]. Exosomes can regulate various physiological and pathological reactions, including tumor cell invasion, cell proliferation and apoptosis, cell differentiation, vascular growth, metastasis, and immune response [4-7]. Furthermore, other studies have demonstrated that exosomes play an important role in organ-specific and non-organ-specific autoimmune diseases, such as systemic lupus erythematosus, rheumatoid arthritis, Sjögren's syndrome, scleroderma, and dermatomyositis [8]. Allergic contact dermatitis (ACD) is an autoimmune disease of unknown cause. A growing number of recent studies have been conducted on the use of exosomes in the regulation, diagnosis, and treatment of ACD. This review offers a look at the progress of research on exosomes in ACD.

## ACD

Contact dermatitis (CD) is a common inflammatory skin disease classifiable into irritant CD (ICD) and ACD [9]. ACD is an allergen-specific, T-cell-mediated inflammatory response dominated by type IV hypersensitivity. The occurrence of ACD requires the exposure of the epidermis to a sensitized contact allergen (Table 1) [10, 11].

Haptens are small allergen molecules with immunoreactivity but no immunogenicity [28]. The contact allergen that causes an allergic contact dermatitis reaction is a hapten. The pathogenesis of ACD is very complex and mainly involves the sensitization and induction stages [28-30]. In the sensitization stage, when the hapten makes contact with the skin, it binds to proteins in the skin to form antigens and obtain immunity [30]. These antigens are recognized by antigen-presenting cells called Langerhans cells (LCs) in the epidermis, swallowed and digested, and then expressed on the surface of the LCs, as an antigen-MHC (major histocompatibility complex). When this antigen-MHC is carried into nearby lymph nodes, it activates T cells to form hapten-specific T cells that differentiate into memory cells and effector T cells and circulate throughout the body [30, 31]. If an individual who is sensitized to a hapten is exposed to the same allergen again, memory T cells are activated, and hapten-specific T cells attract monocyte macrophages and neutrophilic cells to antigen sites in the skin, producing many lymphokines (interleukin-2, interleukin-4, interferon-γ, etc.) that cause inflammatory reactions [30]. This is called the ‘induction phase.’

Although various theories have been proposed for haptens, APCs, and effector T-cells, there is no clear explanation for ACD in patients with specific haptens. Between March 2017 and December 2018, the North American Contact Dermatitis Group (NACDG) performed patch experiments on 4,947 dermatitis patients among whom 2,495 were initially diagnosed with ACD [32]. Nickel accounted for 16.2% as a common allergen, methylisothiazolinone 0.2% aqueous accounted for 15.3% and methylchloroisothiazolinone/methylisothiazolinone 0.02% aqueous accounted for 11.0% [32]. Generally, most people do not respond to exposure-specific haptens.

ACD is classified into acute, subacute, or chronic forms [33]. It occurs in case of skin contact with individuals with advanced sensitivity to allergens [11, 34]. The irritant is usually non-irritating in nature. Acute ACD involves the development of erythema, papules, and vesicles as the most common features [35]. Severe cases manifest bullous pemphigoid. Chronic ACD often presents with erythema and pruritus lesions and may show more numbers of chronic inflammatory spots, such as lichenization, scaling, and fissures [36]. With the breakdown of the epidermal barrier, as reported in chronic ACD, repeated infections can occur [37]. Tinosorb M (10%Petrolatum) is a UV filter, with 87 patients being tested for patches in a cosmetic series, including Tinosorb M (10% Petrolatum), in the Dermatology Department of the University Hospital of Coimbra between February 2009 and April 2012 [38-39]. Five patients aged 39-66 years (three of whom were female and two male) were found to be positive for Tinosorb M in patch experiments [38]. The presence of erythema in the facial and anterior neck positions, associated with pruritus and scaly in duration, still recurred after topical treatment with corticosteroids [38]. Subacute ACD is difficult to describe and can exhibit a variety of features.

According to statistics, the number of people visiting a hospital for consultation due to CD accounts for 4-7% of all visits globally, with a prevalence rate of 15.2% in adolescents and 18.6% in adults. Notably, age had no direct effect on sensitization ability [40]. ICD and ACD share similar clinical phenotypes, which makes it difficult to investigate the prevalence of ACD and precisely determine the number of people who suffer from this disease across the world [40]. ACD seems easy to treat, and all that the patient needs to do is avoid exposure to allergens. However, detection, avoidance of allergens, and follow-up treatment in these cases are challenging. In addition, due to itching, pain, sleep disorders, mental health disorders, and other manifestations of this disease, ACD seriously affects the quality of life of patients, making it a serious socio-economic problem [11]. With the advancements in medical science, people are increasingly gaining awareness of its harm and the importance of therapeutic countermeasures. Finding a treatment for ACD remains a global challenge due to the lack of accurate detection methods and the avoidance of common allergens.

## Exosomes 

Exosomes are formed by the invagination of intracellular lysosomal particles into multivesicular bodies (MVB) that are secreted into the extracellular matrix after fusing the MVB extracellular membrane with the cell membrane. Exosomes can carry specific proteins, RNAs (such as miRNA, lncRNA, circRNAs), and DNA from donor cells [41]. Exosomes can be secreted by a wide variety of cells, including allergic and immune-reactive cells, such as T and B lymphocytes, dendritic cells (DCs), macrophages, and eosinophils, and they are commonly detected in the blood, urine, amniotic fluid, saliva, and other body fluids [42-44].

The types of cells that release exosomes determine the biological functions of the released exosomes. Several studies have demonstrated that exosomes are essential molecules of intercellular communication that are not only involved in triggering downstream signal transduction but also in specifically targeting receptor cells and exchange proteins. Exosomes secreted by fibroblasts loaded with miR-21_3p can be ingested by cardiomyocytes and induce cardiac hypertrophy [45]. Similarly, human keratinocyte-derived exosomes promote coagulation and angiogenesis [6]. Non-small cell lung cancer (NSCLC) cell line HCC827IR-derived exosomes participate in the metastasis of cancer cells [46]. In the adherent-invasive *E. coli* (AIEC) mouse model, breast milk-derived, exosome-loaded oligosaccharides have anti-infectious and anti-inflammatory effects and play an important role in immunomodulation during lactation [47]. Exosomes also play a unique role in various pathogens, such as viruses and prions. Inhibition of exosome secretion using exosome inhibitors reduces cell-to-cell prion transfer, and the relative stimulation of exosome secretion promotes prion transmission [48]. In addition, LPS-stimulated chicken macrophage-derived exosomes can stimulate the immune system and promote the organism's immune response [49]. Exosomes can also provide specific nucleic acid and transport functions. Spherical nucleic acids (SNAs) in PC-3 prostate cancer cells can be coated by exosomes and secreted to the extracellular space. These SNA constructs can then be artificially isolated and re-introduced into the PC-3 prostate cancer cells [50].

## Isolation of Exosomes

Significant progress has been made in the isolation of exosomes over the past few years [51]. Table 2 summarizes and compares the 6 typical exosome separation methods in terms of principles, advantages, and disadvantages.

The lack of standardization of exosome isolation techniques has seriously limited the relevant diagnosis and treatment methods. Lin *et al*. believe that exosome separation technology based on microfluidics has broader application prospects than conventional methods. This approach facilitates the integration of exosome separation and detection functions into a single chip, thereby reducing complexity, time, and cost [85]. Dr. Abramowicz *et al*. proposed that separation technology determines the quality and integrity of exosomes, which are critical factors in the downstream outcomes [86]. The disadvantages of the existing separation technologies, such as interferents in the separation products and the destruction of the exosome structure, can cause misleading results in the downstream analysis, [86].

Wang *et al*. reported a new method for the rapid separation and extraction of tissue exosomes, which involves differential centrifugation, ultrafiltration, size exclusion chromatography (SEC) exclusion, and ultrafiltration [87]. The exudates from the heart, liver, kidney, human colon, breast, and atherosclerotic tissues were observed by transmission electron microscopy, nanoparticle tracing, and tissue isolation [87]. The results revealed that the diameter of the exosome was 40-160 nm and that the structure was evident [87]. The protein secreted by the exocrine tissues was small in size but high in content [87]. As their method combines a variety of separation technologies while meeting the requirements of separation and purification of exosomes, it simplifies the initial steps, eases the operation, and saves enrichment time. The resultant extracted exosomes have fewer impurities and a more comprehensive application range, which is helpful for further exploring significant factors such as the signal pathway mechanism, differential diagnosis, prognosis, and recurrence monitoring of disease-targeted drug delivery therapy [87].

## Role of Exosomes in Allergic Diseases

### Exosomes Involved in Antigen Presentation

DCs are the most functional of antigen-presenting cells (APCs), as their derived exosomes carry complexes with antigen polypeptides on their surface [88]. Such exosomes can act as APC-like substances, and substances bind to antigen MHC-specific receptors on the T-cell surface to induce T cells into playing a role. In a delayed hypersensitivity (DTH) mouse model, DC-derived exosomes were labeled with PKH67 (green fluorescent cell linker) and injected into mice; these fluorescently labeled exosomes were found in the spleen and liver, and CD11c+ DCs and F4/80+ macrophages interacted [89]. Adoptive transfer of CD11c+ or CD3+ splenic T lymphocytes revealed reduced footpad swelling in mice, and DC-derived exosomes may partially inhibit the DTH response by inducing a regulatory T-cell population. Exosomes may be able to regulate the activity of endogenous APCs and T cells [89].

Exosomes secreted by B1 cells possess hapten-specific light chains (LCs) that encapsulate T cells, which can be activated by NKT cells upon skin hapten sensitization. This observation indicates that B1 cells participate in regulating contact hypersensitivity (CHS) responses. In this model, the antigen-recognition system complements inhibitory exosomes to enable the downregulation functions of autoantigen-specific LCs during a CHS response. Multiple neighbors of these LCs may confer enhanced affinity, which results in tighter antigen-specific binding. LCs may be related to the antigen specificity of exosome-mediated CHS inhibition that targets APCs [90].

### Exosomes Transport Inflammation-Related Proteins

Renal collecting duct epithelial cells actively secrete exosomes into the urine, and the purified output from the urinary system contains Leucine-Rich Repeat Kinase2 (LRRK2) protein [91]. LRRK2 influences the occurrence and severity of inflammatory bowel disease through the nuclear factor of activated T cells 1 (NFAT1) activity regulation. Furthermore, the dissemination of this protein occurs through exosomes that are distantly placed from the initial lesion, which is one of the potential mechanisms for increasing neutrophil-mediated inflammation [92].

Superoxide dismutase 2 (SOD2) and glutathione peroxidase 3 (GPX3) are present in exosomes, and both proteins are involved in antioxidant defense [93, 94]. During pro-inflammatory conditions, proteins such as SOD2 and GPX3 are upregulated. SOD2 regulates reactive oxygen species (ROS) levels by activating the peroxide O2 radical (O_2_^-^) and hydrogen peroxide [95]. The transcription factor NF-κB inhibitor suppresses SOD2 production, and reduced SOD2 expression leads to an increased secretion of ROS [94]. We can propose that NF-κB is involved in the expression of SOD2 and that SOD2 can regulate ROS secretion [96]. This dual activity of SOD2 and GPX3 demonstrates that exosomes may contribute to regulating inflammatory levels.

When the nod-like receptor protein 3 (NLRP3) inflammasome is activated, the secreted exosomes trigger the NF-κB signaling pathway, leading to inflammation by mild induction of cellular inflammatory necrosis [97]. Previous studies have shown that IL-1, IL-18, and caspase-1 are involved in response to ACD, and IL-18 plays a vital role in resolving contact allergens and stimulants [98]. Here, we propose that the presence of NLRP3 in the skin is necessary to sense the invasion of pathogenic bacteria and activate the NLRP3 inflammasome. It is further essential to trigger the maturation and release of downstream IL-1 and IL-18, which in turn respond specifically to T cells in ACD modulation.

### Exosomes Promote the Release of Inflammatory Mediators

Bretz *et al*. isolated exosomes from amniotic fluid, cirrhotic ascites, and ovarian cancer patients [99]. Their study revealed that exosomes trigger NF-κB and STAT3 activation through Toll-like receptor signaling, promoting the secretion of inflammatory cytokines in monocytes. Further, it showed that regardless of cancer-associated body fluids, the exosomes induced the secretion of pro-inflammatory mediators [99]. Studies have also indicated chemokine chemoattractant protein-2 (CCL2) inflammation in exosomes of mice with acute and chronic kidney injury, suggesting a link between inflammation and kidney disease. CCL2 increases in exosomes after treatment with renal tubular epithelial cells, leading to the appearance of inflammation. Upon bovine serum albumin (BSA) treatment of tubular epithelial cell-derived exosomes through tail vein injection, tubular damage and renal inflammation were observed in the mice model [100].

As mentioned earlier, the mechanism of ACD pathogenesis can be categorized into two stages: sensitization and induction. In the sensitization phase, allergen stimulates APC activation and maturation, and the APCs present the antigen complexes to T cells [28]. Also, in this stage, APCs stimulate keratinocytes into releasing the inflammatory factor tumor necrosis factor-alpha (TNF-α) and interferon-gamma (IFN-γ), thereby enabling T cells to recognize antigenic peptides [30, 31]. During the induction phase, the skin is again exposed to allergens that induce skin injury by producing large amounts of inflammatory cytokines [28]. Moreover, inflammatory signals are enhanced by T cell, NK cell, and macrophage activation, thus promoting tissue damage. Guo *et al*., using mesenchymal stem cell (MSCs)-derived exosome intravenous ACD-model mice, observed decreased ear swelling, leukocyte infiltration, decreased TNF-α and IFN-γ, and elevated anti-inflammatory factor IL-10 in the treated group [101]. Therefore, we can speculate that exosomes isolated from ACD patients may stimulate the release of inflammatory mediators.

### Exosomes Transport Mitochondria Involved in Pro-Inflammatory Signaling

Mitochondria are known as inflammatory sensors because they can produce ROS and participate in the inflammatory pathway [102]. According to recent research, the presence of mitochondria is noted in exosomes of proinflammatory human leukocyte antigen DR+ (HLA-DR+) subsets of airway myeloid-derived regulatory cells [103]. Myeloid-derived regulatory cells (MDRCs) are the known regulatory factors of T-cell response in asthma, and the HLA-DR+ human MDRC subset produces ROS and promotes CD4+ T-cell proliferation. Dr. Hough and colleagues cocultured the MitoTracker Green (MitoT-Green)-labeled bronchoalveolar lavage (BAL) exosomes with the peripheral blood T cells and observed the MitoT-Green+ exosomes within the T cells [103]. The MDRCs were labeled with MitoT-Green, and then the MDRC-derived exosomes were cocultured with the peripheral blood T cells, again observing the MitoT-Green+ exosomes within the T cells. It indicates that the mitochondria can transfer from these exosomes into the T cells. Cocultured peripheral T cells with MDRCs with different-colored MitoTracker, and labeled, MDRC-derived exosomes and peripheral T cells revealed that these exosome-transferred mitochondria merged with T-cell mitochondria and produced ROS [103]. The ROS produced by exosomal-transported mitochondria can further aggravate inflammation, impair the airway epithelium, and exacerbate asthma [103].

Nickel (Ni), an environmental pollutant, is commonly used in the manufacturing of electronic equipment and the medical industry [23, 104]. Individuals in immediate contact with it are affected by allergic and hypersensitivity reactions. Ni^2+^ ions induce mitochondria, generate ROS, release mitochondrial DNA, and activate the NLRP3 inflammasome pathway [105]. Hence, we can hypothesize that the ROS produced by exosomes transports mitochondria in ACD patients and promotes inflammation as well as the severity of allergic reactions.

## Function of Exosomes in ACD Pathogenesis

The pathogenesis of ACD is complex, and it is connected to cellular immunity and the molecular inflammatory network. Guo *et al*., using the CHS mouse model, established that exosomes could inhibit the development of Th1 and T cytotoxic type 1 (Tc1) cells and reduce the secretion levels of IL-1β, TNF-α, and IFN-γ. Exosomes also promoted the expression of Treg and increased the levels of IL-10 [101]. MiRNAs are the main functional components of exosomes, and they can regulate the genetic information in receptor cells by regulating the downstream mechanisms of the STAT1 pathway [106]. The crucial miRNAs involved in this process are miRNA-146a, miRNA-181a, and miRNA-150. In the mouse melanoma model, miRNA-146a is regulated by STAT1 and STAT1 cytokine interferon-γ, reducing cell migration, cell cycle activity, and oxygen consumption. Previous reports suggest that in the JAK/ STAT pathway, overexpression of miRNA-181a and miRNA-150 inhibits the effects of DCs and inflammatory response. Furthermore, studies by Guo *et al*. mentioned that the STAT1 signal is inhibited, suggesting that it can affect the regulation of CD3+ T cells, which is consistent with the above conclusion [107].

## Role of Exosomes in Diagnosis

ACD can be diagnosed through routine laboratory procedures such as blood analysis and biochemistry [40]. The presence of acidic granulosa cells during blood microscopic analysis, in addition to altered liver and kidney function tests during blood biochemistry and routine urine testing indicate ACD during patient diagnosis. The patch test, the primary way to diagnose ACD, can also be carried out later [107]. It is a simple procedure that includes several layers of gauze folded into a specific size, moistened with the test substance, and placed in close contact. The positive results are observed as a local reaction after 24 or 48 h [107].

MiRNAs, an important aspect of exosomes, play an essential role in regulating gene expression. MiRNAs are non-coding regulatory RNAs of approximately 22 nucleotides in length [41]. Exosomes are, to some extent, containers for miRNAs. Earlier reports suggested the upregulated expression of miRNA-21, miRNA-223, miRNA-142-3p, and miRNA-142-5p, in the skin of diphenylcyclopropenone (DPCP) and dinitrofluorobenzene (DNFB)-sensitized subjects, but not in the non-sensitized mice [108]. Thus, these four miRNAs play a role in adaptive response. The miRNAs that were elevated were miRNA-21, miRNA-223, miRNA-142-3P, and miRNA-142-5P, and these were implicated in T-cell activation [108]. ACD is a disease mediated mainly by CD8+ T cells and Th1 cells. The study showed that miRNAs might participate in the pathogenesis of ACD. Further studies to develop a miRNA map specific to ACD can aid in its diagnosis [108].

## Therapeutic Effects of Exosomes from Different Sources on ACD

Several cell types can secrete exosomes in normal and pathological states. Some examples are DCs, T and B lymphocytes, epidermal cells, mesenchymal stem cells (MSCs), adipocytes, and platelets. Currently, MSCs and DC-derived exosomes are reported to be able to treat ACD.

## Therapeutic Efficacy of Mesenchymal Stem Cells

MSCs are non-hematopoietic, pluripotent stem cells with the potential for self-renewal and pleiotropic differentiation [109]. They also have vast potential for tissue repair and immune regulatory functions. MSCs are chemotactic, migrating to damaged tissue when local inflammation occurs. This process can complete tissue repair through direct differentiation or produce immune factors to regulate the inflammatory tissue microenvironment. MSCs can further promote the release of immunosuppressive and growth factors, thus leading to endothelial cell angiogenesis and extracellular matrix remodeling [109, 110].

The therapeutic efficacy of multipotent MSCs has been demonstrated in various disease models through prior studies. Further, Golubinskaya investigated the application of MSCs to exosomes in ACD [111]. The cream formula prepared using MSC exosomes and dexamethasone worked better than fluticasone for ACD treatment. Also, the lymphocyte infiltration rate was significantly reduced in the exosome group compared to the glucocorticoid group [111]. Secretory MSCs have anti-inflammatory effects and are known to reduce the skin inflammatory response. However, further studies are needed to explore the therapeutic mechanisms underneath this process.

Guo *et al*. found that MSC-derived exosomes (MSC-Exo) can act directly on CD3+ T cells and inhibit pro-inflammatory factors such as IFN-γ and TNF-α. They can lead to the differentiation of Th1 and Tc1 cells while also promoting the differentiation of Treg cells and the expression of anti-inflammatory factor IL-10 [106]. In addition, it was speculated that MSC-Exo might mediate miRNA-regulated STAT1 and pstat1 protein expression at post-transcriptional and post-translational levels, which may affect T-cell immunity [112]. Thus, the study provides a new preclinical basis for using MSC-Exo in treating ACD-related diseases.

## Therapeutic Efficacy of Dendritic Cells

The indoleamine-pyrrole 2,3-dioxygenase (IDO) enzyme is an immune tolerance regulator capable of inducing the differentiation of regulatory T cells and controlling allergy-associated Th2 inflammation. Tryptophan is an amino acid essential for T-cell activation, and IDO inhibits T cells by consuming tryptophan [113, 114]. This makes T cells unable to produce specific immune effector cells and specific antibodies so they are incapable of performing a normal immune response. IDO is also a key element in the induction of immune tolerance [115].

Among people sensitive to allergens, those with strong IDO enzymes have higher IDO activity than those with severe reactions. Previous studies have shown that exosomes produced by DCs that were treated with adenovirus vectors expressing FasL, IL-10, or IL-4 could inhibit the DTH mice model [116]. Dr. Bianco and colleagues proposed that bone marrow (BM)-derived DCs transduced with IDO or CTLO-4Ig (IDO inducer) can inhibit DTH mouse models [116]. The DCs of IDO exosomes had anti-inflammatory effects, and their inhibitory effects depended partly on B7 co-stimulatory molecules [116]. Furthermore, the transfer of the CTLA4Ig to DCs caused the induction of IDO in DCs and exosomes to reduce inflammation in an IDO-dependent manner. This indicates that DC-derived exosomes can inhibit immune responses, reduce cellular inflammation, and are a good therapeutic option for treating immune diseases such as ACD and other inflammatory conditions.

In summary, the occurrence and development of ACD may enlist exosomes, which may play a certain role in the diagnosis and treatment of ACD. Although the functions of exosomes have been extensively studied, some of their physiological functions and mechanisms remain unknown. Presently, the research on the effect of exosomes on ACD is at its initial stage and the molecular mechanism of exosomes involved in ACD remains unclear. The role of specific proteins, RNA, and DNA in ACD in different source exosomes warrants further investigation, and the signal transduction pathway and inflammatory cascade process of their cytokines in ACD need to be further explored. Research into exosomes is an emerging field of ACD. Under the research model of translational medicine, it can significantly promote the progress of ACD research and bring new opportunities for clinical diagnosis and treatment.

In other words, exosomes are the focus of domestic and foreign research at the present time. With continuous exploration and discovery, the constant improvement of bioinformatics, high-throughput sequencing technology, and genomics can undoubtedly provide new progress in the diagnosis, treatment, and clinical application of exosomes in ACD.

## Figures and Tables

**Table 1:** Partial contact allergens causing contact allergic dermatitis.

Contact allergen	Source	Reference
2-Hydroxyethyl salicylate	Veterinary anti-inflammatory gel	[12]
Acrylates	Dental materials, artificial nails	[13]
Aliphatic polyisocyanates	Shin pads, footwear, and other types of sports equipment based on ethylene vinyl acetate copolymers	[14]
Bisabolol	Moisturizers	[15]
Butylhydroxytoulene	Cosmetics	[16]
Cetyl alcohol	Emulsifiers in many consumer products and topical medications	[17]
Cosmetic Blush	Cosmetics	[18]
Dimethyl fumarate	Furniture, shoes, textiles, etc.	[19]
Majantol	Fragrances	[20]
Methylene bis-benzotriazolyl tetramethylbutylphenol	Sunscreens	[21]
Methylchloroisothiazolinone and Methylisothiazolinone	Cosmetics	[22]
Nickel	Coins, tools, pant snaps, cosmetics, etc.	[23]
Octylisothiazolinone	Biocide	[24]
Peppermint Oil	Lip balm	[25]
p-Phenylenediamine	Permanent hair-coloring agent or oxidative hair dyes	[26]
Sorbitans	Topical corticosteroid formulations	[27]

**Table 2:** Comparison of exosome isolation methods.

Methods		Theory	Strengths	Weaknesses	Reference
Isolation technology based on super centrifuge	differential ultracentrifugation	Differences in density, size, and quality	Exosomes with similar density but different sizes were isolated	Predisposition to contamination and exosome loss	[52-54]
	density gradient ultracentrifugation	Differences in size and quality	Easy to recycle	Capacity is limited by the narrow loading area	[55-58]
Isolation technology based on sieving size	ultrafiltration	Molecules of different sizes or molecular weights	Fast speed, high purity, no need for special equipment	The influence of external force is large	[59-63]
	size exclusion chromatography (SEC)	Depending on the size of the macromolecules and particles	Minimal change in exosome characteristics	Difficult to operate	[61, 62-69]
	flow field-flow fractionation (F4)	The molecules of different sizes	Rapid isolation and characterization of exosomes	/	[2, 70-71]
Isolation technology based on polymer precipitation		Change solubility and decomposition	Simple operation, no need for special instruments	Low purity, easy to mix with impurities, need to remove impurities	[72-73]
Isolation technology based on microfluidic		Physical and biochemical properties on a microscale	Fast, cheap, automatable, high quality	Lack of standard and large-scale testing	[74-78]
Isolation techniques based on artificial antibodies		Molecular recognition between antigens and antibodies	It is easy to prepare, economical, and suitable for largescale use	The specialty is strong, and the kinds of ligands need to be developed.	[79-81]
Isolation technique based on immunoaffinity capture		The immunological affinity between antigenic antibodies	High specificity and high purity	High price, the antibodies can be blocked, the antibody membrane is specially made, the steps are tedious	[82-84]
